# Evaluating the Implementation of a Mobile Phone–Based Telemonitoring Program: Longitudinal Study Guided by the Consolidated Framework for Implementation Research

**DOI:** 10.2196/10768

**Published:** 2018-07-31

**Authors:** Patrick Ware, Heather J Ross, Joseph A Cafazzo, Audrey Laporte, Kayleigh Gordon, Emily Seto

**Affiliations:** ^1^ Institute of Health Policy, Management and Evaluation Dalla Lana School of Public Health University of Toronto Toronto, ON Canada; ^2^ Centre for Global eHealth Innovation Techna Institute University Health Network Toronto, ON Canada; ^3^ Ted Rogers Centre for Heart Research University Health Network Toronto, ON Canada; ^4^ Department of Medicine University of Toronto Toronto, ON Canada; ^5^ Peter Munk Cardiac Centre University Health Network Toronto, ON Canada; ^6^ Institute of Biomaterials and Biomedical Engineering University of Toronto Toronto, ON Canada; ^7^ Canadian Centre for Health Economics Toronto, ON Canada

**Keywords:** Consolidated Framework for Implementation Research, eHealth, heart failure, implementation, telemonitoring

## Abstract

**Background:**

Telemonitoring has shown promise for alleviating the burden of heart failure on individuals and health systems. However, real-world implementation of sustained programs is rare.

**Objective:**

The objective of this study was to evaluate the implementation of a mobile phone–based telemonitoring program, which has been implemented as part of standard care in a specialty heart function clinic by answering two research questions: (1) To what extent was the telemonitoring program successfully implemented? (2) What were the barriers and facilitators to implementing the telemonitoring program?

**Methods:**

We conducted a longitudinal single case study. The implementation success was evaluated using the following four implementation outcomes: adoption, penetration, feasibility, and fidelity. Semistructured interviews based on the Consolidated Framework for Implementation Research (CFIR) were conducted at 0, 4, and 12 months with 12 program staff members to identify the barriers and facilitators of the implementation.

**Results:**

One year after the implementation, 98 patients and 8 clinicians were enrolled in the program. Despite minor technical issues, the intervention was used as intended. We obtained qualitative data from clinicians (n=8) and implementation staff members (n=4) for 24 CFIR constructs. A total of 12 constructs were facilitators clustered in the CFIR domains of inner setting (culture, tension for change, compatibility, relative priority, learning climate, leadership engagement, and available resources), characteristics of individuals (knowledge and beliefs about the intervention and self-efficacy), and process (engaging and reflecting and evaluating). In addition, we identified other notable facilitators from the characteristics of the intervention domain (relative advantage and adaptability) and the outer setting (patient needs and resources). Four constructs were perceived as minor barriers— the complexity of the intervention, cost, inadequate communication among high-level stakeholders, and the absence of a formal implementation plan. The remaining CFIR constructs had a neutral impact on the overall implementation.

**Conclusions:**

This is the first comprehensive evaluation of the implementation of a mobile phone–based telemonitoring program. Although the acceptability of the telemonitoring system was high, the strongest facilitators to the implementation success were related to the implementation context. By identifying what works and what does not in a real-world clinical context using a framework-guided approach, this work will inform the design of telemonitoring services and implementation strategies of similar telemonitoring interventions.

## Introduction

Heart failure telemonitoring systems are developed with the objective of reducing mortality and hospitalizations and improving patients’ quality of life [1]. However, despite patient acceptance [2-4], the diffusion of these services is lagging [5]. Meta-analyses tend to support claims of the positive impact on heart failure outcomes [1,6-9]; however, important inconsistencies in the evidence persist [10]. Inconsistencies are believed to result from the heterogeneity of the intervention and patient populations used in primary studies and the lack of consistency with which interventions are used (ie, fidelity) in clinical trials [10]. Much remains to be learned on how to best implement telemonitoring interventions such that true effectiveness can be measured and their benefits be fully realized.

Systematic reviews present extensive lists of various barriers and facilitators to telemonitoring implementation [11-13]. External barriers include the lack of a clear business model in single-payer health systems [14] and the lack of acceptable reimbursement methods for clinician users [15]. Furthermore, clinician adoption is influenced by the quality and usability of the technology, compatibility of the intervention with existing work processes, and intrinsic clinician motivation to adopt telemonitoring as part of their practice [11-13]. In fact, a recent review found that the challenge presented by new and often ill-defined clinician roles within changing workflows was a key factor in leading to the failure of eHealth interventions [16]. One multisite qualitative study similarly highlights the importance of contextual factors for clinician adoption, including the degree of support clinicians receive in their new roles and alignment with organization objectives [17]. However, most telemonitoring implementation studies have been conducted retrospectively, which does not allow for a robust analysis of how these barriers and facilitators exert their influence over the entire implementation period. In addition, few studies report on quantitative outcomes to justify judgments of the implementation success or failure.

A mobile phone–based telemonitoring program called *Medly* was implemented as part of the standard of care at a specialty heart function (HF) clinic in Toronto, Canada. This program features a system that has previously demonstrated improvements in clinical outcomes, patient self-care, and quality of life [18]. The objective of this study was to evaluate the implementation of the *Medly* program by answering two research questions: (1) To what extent was the *Medly* program successfully implemented? (2) What were the barriers and facilitators to implementing the *Medly* program?

## Methods

### Study Design

This study used a longitudinal single case study design. The case was defined as the telemonitoring intervention and the implementation site as described below for one year following the enrollment of the first patient (August 23, 2016). The units of analysis for this evaluation were the Ted Rogers Centre of Excellence for Heart Function (HF clinic) and program staff with data for determining the implementation success being collected through a document review. In addition, barriers and facilitators were assessed using semistructured interviews with program staff guided by the Consolidated Framework for Implementation Research (CFIR) [19]. Notably, the patient perspective, including reasons for use, adherence, and withdrawal will feature in an upcoming publication. In this study, data collection was conducted within the context of a larger quality improvement program evaluation [20], which has been approved by the University Health Network (UHN) Research Ethics Board (16-5789).

### Intervention

The *Medly* program consists of two components: (1) the technology (hardware and software) and (2) the human-dependent interactions and services.

#### Medly Technology

The patient-facing technology includes the *Medly* mobile phone app, which works by allowing patients with heart failure to record the following three parameters: (1) weight; (2) blood pressure; and (3) symptoms. Based on these data inputs by patients at home, the *Medly* app, which contains a rule-based algorithm customized according to patient-specific target ranges, displays self-care messages and generates alerts that are automatically relayed to a clinician when signs of clinically significant health status deterioration occur. Patients were instructed to record the three parameters daily, and they would receive an automated phone call if they had not done so before 10 am; this was intended to assist with compliance. For the launch of the program, each patient was provided with all the required equipment, which includes a mobile phone with a data plan, a Bluetooth-enabled weight scale, and a Bluetooth-enabled blood pressure monitor.

The clinician-facing technology seeks to support the management of the patient alerts; this is primarily conducted through a Web-based interface (ie, the dashboard) containing a list of patient alerts, graphs showing patient-level trends of the three clinical parameters monitored, and heart failure-specific lab results. In addition, clinicians have the option of receiving alerts through automated emails, which contain the latest weight, blood pressure, and symptoms. Furthermore, the email contains the patients’ current medication list, heart failure-related laboratory results, and contact information.

#### Medly Services

Enrollment into the program was based on clinical judgment. After discussing the program with patients, a clinician, ie, a cardiologist, a nurse practitioner (NP), or a resident, fills out a form to indicate the desired target ranges needed to customize the algorithm. Then, a telehealth analyst (THA) provides patients with the *Medly* technology and training on how to use it. When alerts are triggered, they are viewable by patients’ treating cardiologist and NPs. The clinicians might act independently or communicate among themselves by email or in person to determine the best course of action. If required, a clinician will follow up with patients either by phone or email, documenting all actions and decisions in the hospital electronic medical record (EMR). Furthermore, patients and clinicians are instructed to contact the THA to receive the technical support, if required.

### Implementation Site

The HF clinic, part of UHN, is a high-volume specialty care clinic for patients with heart failure in Toronto. The intervention was developed by UHN’s Centre for Global eHealth Innovation (eHI) in close collaboration with clinicians from the HF clinic. The THA is employed by the UHN Telehealth Department with 25% of their time dedicated to supporting the *Medly* program. The HF clinic, UHN telehealth services, and eHI are physically located in the same building.

### Implementation Strategy

Preparations for the program launch included the development of training materials for patients (user manual and training checklist). In addition, clinician users were provided with a user guide and a training session lasting approximately 1 hour. Moreover, members of the eHI team followed a service design methodology, consisting of mapping clinic workflows and producing a service blueprint for the *Medly* program, which sought to minimize the disruption to existing HF clinic processes.

### Implementation Outcomes

We selected 4 implementation outcomes from Proctor et al’s Implementation Outcomes Framework as measures of the implementation success [21]. In addition, data on the outcomes, defined below, were collected after 4 and 12 months of the launch through a document review process and semistructured interviews.

- *Adoption*: The number of clinicians deciding to monitor patients using the *Medly* system.
- *Penetration:* The level of integration of the *Medly* program within the existing services of the HF clinic.
- *Feasibility:* The extent to which the *Medly* program can be successfully used by patients.
- *Fidelity:* The extent to which the *Medly* program is being used as initially intended.

### Barriers and Facilitators to Implementation

Semistructured interviews were developed based on the constructs of the CFIR, which provides a pragmatic organization of theory-informed constructs known to impact the implementation success across the following 5 domains: (1) intervention characteristics; (2) outer setting (eg, patient needs and resources, external policy and incentives, etc); (3) inner setting (networks and communication, implementation climate, readiness for implementation, etc); (4) characteristics of individuals; and (5) process [18]. Further interview probes were developed to explain the quantitative implementation outcome indicators. Moreover, interviews were conducted prior to the program launch, and again after 4 and 12 months, each session lasting 30-60 minutes. Of note, all adopting clinicians and eHI *Medly* program staff were invited for participation. In addition, clinicians who had not adopted the system by 12 months were also invited to participate. All interviews were recorded and transcribed for later qualitative analysis.

### Data Analysis

The interview transcripts were analyzed by two independent investigators (PW and KG) using the Framework Method [22]; this involved a largely deductive thematic analysis using a codebook based on the CFIR constructs [19]. PW and KG independently coded the transcripts and then met to discuss contradictory codes and passages. The management of source documents and coding was done with the help of NVivo version 11 (QSR International, Doncaster, Victoria, Australia). To determine the degree to which the barriers and facilitators impacted the implementation, valence ratings were attributed by PW and KG to each construct according to the criteria outlined by Damschroder et al [23]. Qualitative findings and valence ratings were validated during a meeting with key members of the clinician and eHI program staff (n=6).

## Results

### Study Participants

In this study, 8 clinicians participated. One cardiologist, who was the only clinician who had not adopted the technology before the end of the study period, did not respond to requests to be interviewed prior to completion of the manuscript. Table 1 shows the interview schedule and the role of each participant.

### Implementation Outcomes

Table 2 presents the results of implementation outcomes, which are discussed below.

#### Adoption and Penetration

The program was launched with 3 clinician users (1 cardiologist and 2 NPs). By the 12-month time point, 5 additional cardiologists were monitoring patients using *Medly*, representing an increase in the penetration of the *Medly* program in the HF clinic from 38% to 89%, a diffusion pattern that is explained in the interviews.

**Table 1:** Study participants and timing of interviews. An "X" incicates an interview was conducted at the specified time point.

Study identifier	Role in the program	Role descriptor	Baseline	4 months	12 months
Clinician 1	Cardiologist and clinical lead of the Ted Rogers Centre of Excellence for Heart Function	Early adopter		X	X
Clinician 2	Nurse practitioner	Early adopter	X	X	X
Clinician 3	Nurse practitioner	Early adopter	X	X	X
Clinician 4	Cardiologist	Late adopter (9 months)			X
Clinician 5	Cardiologist	Late adopter (11 months)			X
Clinician 6	Cardiologist	Late adopter (11 months)			X
Clinician 7	Cardiologist	Late adopter (11 months)			X
Clinician 8	Cardiologist	Late adopter (11 months)			X
eHealth 1	Project manager	Left on maternity leave after 4 months	X		
eHealth 2	Project manager	Replaced original project manager		X	X
eHealth 3	Program operations lead	New position was created after 3 months		X	X
eHealth 4	Telehealth analyst			X	X

**Table 2:** Implementation outcomes indicators.

Implementation outcome^a^ and indicator			4 months		12 months
**Adoption**					
	Number of clinicians having decided to use *Medly* to monitor patients	3		8	
**Penetration**					
	Percentage of clinicians using *Medly* over the total number of potential clinician users in the Ted Rogers Centre of Excellence for Heart Function	38		89	
**Feasibility**					
	Cumulative number of patients enrolled in the *Medly* program	42		98	
	Cumulative number of patients removed from the *Medly* program for clinical reasons (eg, received a heart transplant)	0		8	
	Cumulative number of patients having chosen to leave the *Medly* program	0		5	
	Number of deaths (all unrelated to the program)	1		5	
**Fidelity**					
	Cumulative number of calls or emails made to the telehealth analyst for technical assistance	56		195	
	Cumulative number of requests for changes to the *Medly* technology	15		72	

^a^Implementation outcomes are defined in the framework by Proctor et al [21].

All participants described how the *Medly* program was initially only open to the 3 clinicians who were most actively involved in its development. By the 11^th^ month of the program, the clinical lead of the HF clinic decided to open its availability as a resource to other cardiologists. Many of the later adopting clinicians had always expected to be involved and were simply waiting to be invited.

I had no involvement a year ago, I was aware of it, and very supportive of it... [The request] probably came through [Clinician 1] finally saying “we’re at a mature point, Medly is really working, we have good capacity, let’s let the others in…I was just waiting to see when it would happen.

Clinician 7

Although all cardiologists who adopted *Medly* after the initial launch had a similar perspective, some were concerned about the time it would add to their workday. They ultimately decided to participate because they felt a responsibility to share in the workload being taken on by their colleagues. Another important factor swaying their decision was a concern that they could be excluded from their patients’ circle of care.

I’d like to be more involved but I also like to know that I have the time… I think [the reason I decided to participate is] just a sense of fairness. I think it’s just not fair for one person to take over the ownership of it. Again, that speaks to the sustainability. It’s not sustainable for one physician or one nurse or one healthcare professional to be remote monitoring all the data and all the patients all the time…In this case, it’s a cardiologist that I know and trust very well…But again, you don’t want to be left outside the circle of care for a patient that is your patient and your responsibility.

Clinician 5

#### Feasibility

By 12 months, 98 patients were enrolled in the *Medly* program; this was a lower number than initially anticipated and is partially explained by a low initial penetration within the clinic. In addition, throughout the implementation, clinicians began to realize that patients benefited differently depending on their disease severity, ability to use the technology, ability to adhere to taking measures, and receptivity to self-care messages, which led to clinicians becoming more selective of which patients were enrolled.

I also think and I respect that they’re doing their due diligence…about actually finding the right patients. The clinicians need to make sure they’re only targeting patients that would benefit and not someone that they’ll just take off after a week…So of course, it’s a little difficult on their side. They have to do a lot, you have to think a lot more about it. But I feel like they’re being more mindful about it.

eHealth 4

Feasibility is also demonstrated by the relatively low number of patients who chose to stop using the system (n=5). Additionally, 5 patients passed away during the evaluation period. These deaths were determined to be unrelated to the *Medly* program and were explained by clinicians as being reflective of the severe disease state of the patient population.

#### Fidelity

Overall, the intervention is generally being used as intended with clinicians reviewing all alerts generated by *Medly*, following up with patients when necessary, and documenting all actions in EMR. The *Medly* program was launched with the idea that both the system and the service would continue to improve and evolve over time. Throughout the implementation, the THA received 159 calls from patients and 36 calls from clinicians related to problems with the system (eg, receiving adherence calls when they had taken their readings, usability issues, and general connectivity problems between the phone and the peripheral Bluetooth equipment), all representing examples of when the system was not working as intended. However, these, as well as the 72 documented feature requests by patients and clinicians, are evidence of a properly functioning quality improvement mechanism.

### Barriers and Facilitators of Implementation

Table 3 describes the barriers and facilitators of the implementation along with a valence rating signifying the degree to which it had an impact on the implementation of the *Medly* program. Unless otherwise discussed, valences were relatively consistent throughout the entire 12-month implementation period.

#### Intervention Characteristics Domain

##### Evidence Strength and Quality

Clinician participants acknowledged ambiguity in the literature of the impact of telemonitoring for heart failure. However, this did not impact the implementation for reasons identified in the construct *knowledge and beliefs about the intervention*.

##### Relative Advantage

The *Medly* program was perceived as having a relative advantage over alternative telemonitoring options. Unlike many telemonitoring systems, *Medly* measures multiple clinical parameters and offers algorithm-based self-care instructions with structured telephone support when necessary.

It’s another version of what other people have tried. It has more elements than just daily weight because I think we know that daily weights are inadequate for measuring the state of somebody’s heart failure. It can [also] be used with other things, structured telephone follow up as needed. I think the interactions with the nursing staff are an important value add related to Medly.

Clinician 5

##### Adaptability

Many statements revealed the adaptability of both the technology and service components of the *Medly* program, giving this a strong positive valence. Examples include flexibility in how clinicians perform program-related tasks (eg, documentation), workflow adaptations to make more efficient use of the THA’s time, and changes to the *Medly* algorithm.

Pulling us out [of the clinic] was a good change. That’s more to the workflow...When it comes to the actual product, there have been changes, well a lot of feature requests to change the algorithm or to change some copy of the alert and things like that… There are multiple examples of how algorithm changes have already been made and that has helped.

eHealth 4

##### Complexity

Several statements revealed the complex nature of the *Medly* program, giving this construct a negative valence in the early stages of the implementation. Examples of complexity include (1) need for extensive documentation, (2) relying on engagement from patients, (3) challenges in identifying program candidates, (4) communicating patient information between *Medly* clinicians, and (5) setting algorithm parameters.

The biggest time for me is having to create all these communication notes in [the EMR] to document my conversations with people.

Clinician 3

The patient also has an almost 50/50 responsibility. They don’t have to be there when I call, but if I leave a message and if I leave a call back number...I expect that someone is going to call me back.

Clinician 3

There is no literature out there to clearly say who’s the right person...I mean people that I’ve learned are more challenging are people with cognitive impairment and people who, for a variety of reasons, aren’t engaged enough to respond when we contact them.

Clinician 3

**Table 3:** Valence ratings assigned to Consolidated Framework for Implementation Research constructs.

Domains and constructs^a^				Operational definition^a^		Rating assigned to construct^b^
**I. Intervention characteristics**						
	Evidence strength and quality			Perception of the quality and validity of the evidence supporting the use of telemonitoring for heart failure.		0
	Relative advantage			Perception of the advantage of implementing the *Medly* program versus an alternative solution.		+2
	Adaptability			The degree to which the *Medly* program can be adapted to meet the needs of the HF clinic^c^.		+2
	Complexity (reverse rated)			Perceived complexity of the *Medly* program as reflected by the degree of disruptiveness to existing workflows and number of steps involved in using the intervention as intended.		–1
	Design quality and packaging			Perceived quality of the *Medly* program (technology and service components) and how well these components are bundled and work together.		0
	Cost			Financial and opportunity costs of implementing the *Medly* program.		–1
**II. Outer setting**						
	Patient needs and resources			The degree to which heart failure patients’ needs are known and prioritized by the HF clinic (ie, patient-centeredness).		+2
	External policy and incentives			Policies and incentives that support or hinder the implementation of telemonitoring programs.		0
**III. Inner setting**						
	Networks and communications			The quality of the communication networks that support the implementation and daily operations of the *Medly* program.		–1
	Culture			Norms and values of the HF clinic and UHN^d^.		+2
	**Implementation climate**					
		Tension for change	The degree to which stakeholders perceive a need for change in the clinical management of patients in the HF clinic.		+2	
		Compatibility	The degree of fit between the *Medly* program and the HF clinic’s values, norms, needs, and existing workflows and systems.		+1	
		Relative priority	Stakeholders’ perception of the importance of implementing the *Medly* program.		+2	
		Learning climate	The degree to which the HF clinic and UHN have a climate that provides time and space for reflective thinking and that allows team members to feel essential, valued, and safe to try new methods.		+2	
	**Readiness for implementation**					
		Leadership engagement	Commitment, involvement, and accountability of the HF clinic lead.		+2	
		Available resources	The level of resources dedicated for the implementation and ongoing operations of the *Medly* program.		+2	
		Access to knowledge and information	Ease of access to digestible information and knowledge about the *Medly* program and how to incorporate it within existing HF clinic workflows.		0	
**IV. Characteristics of individuals**						
	Knowledge and beliefs about the intervention			Clinicians’ attitudes toward and value placed on the *Medly* program.		+2
	Self-efficacy			Clinicians’ and telehealth analyst’s belief in their own capabilities to execute their role within the *Medly* program and achieve implementation goals.		+2
**V. Process**						
	Planning			The degree to which a plan for implementing the *Medly* program was developed in advance and the quality of that plan.		–1
	**Engaging**					
		Opinion leaders	Individuals in the HF clinic who have a formal or informal influence on the attitudes and beliefs of their colleagues with respect to implementing the *Medly* program.		+1	
		Champions	Individuals who dedicate themselves to supporting and overcoming barriers to the implementation of the *Medly* program.		+2	
	Executing			Carrying out or accomplishing the tasks needed to support the implementation of the *Medly* program.		0
	Reflecting and evaluating			Feedback about the progress and quality of the implementation along with regular debriefing about progress and experience.		+2

^a^The constructs and operational definitions are adapted from the Consolidated Framework for Implementation Research [19].

^b^Definitions of valence ratings are adapted from Damschroder et al’s study [23]: –2, the construct had a strong negative influence on the implementation effort; –1, the construct had a minor negative influence on the implementation effort; 0, the construct had a neutral influence on the implementation effort. Alternatively, different aspects of the construct had a positive influence, while others had negative influence: +1, the construct had a minor positive influence on the implementation effort; +2, the construct is a strong positive influence on the implementation effort.

^c^HF clinic: Ted Rogers Centre of Excellence for Heart Function.

^d^UHN: University Health Network.

If [another clinician], for example, has had to deal with several alert-related issues for a particular patient, how is that information then communicated over to me…if there's some issue I have to follow up on?

Clinician 2

[Another challenge] is this idea of trying to guess what your range of variation is going to be for each patient [when setting parameter thresholds for the algorithm]. I think there are probably more accurate mathematical models to try to come up with that rather than me sort of flipping a coin and deciding, “Okay, it’s going to be 3 pounds + or –, or 2.5 pounds.”

Clinician 6

As these challenges were identified and solutions to mitigate their impact implemented, the complexity of the program was no longer viewed as negatively impacting the program’s continued growth.

##### Design Quality and Packaging

General satisfaction with the *Medly* program and its design were a pervasive theme throughout the interviews. However, some design-related factors were perceived as barriers to continued growth of the program and sustainability even if they did not significantly impact clinician adoption. For example, clinicians expressed a desire for a more seamless integration with existing technologies and workflows by (1) integrating the dashboard with the hospital EMR to facilitate documentation, (2) making the dashboard available on mobile devices, (3) providing more patient details in the dashboard, and (4) allowing for multiple patient-clinician communication modalities (eg, short message service text messaging).

I really wish it worked on my iPhone or my iPad, particularly in the odd time where [Clinician 1] has been away and I’ve had to do it on the weekend, you know, or [when] I needed to check something when I was trapped in New York and I couldn’t work remotely because I can’t get it on my phone.

Clinician 3

I find dashboard right now is very intuitive. I think the part that I find really challenging is that I have to usually have [the EMR] open with Dashboard and so it would be nice to have some patient information in the Dashboard so that you’re not having to go back all the time between those two platforms. The other thing I would say, they’re minor things but you know, contact information for patients. Not simply just one phone number but also an email at the top or the ability to text a patient or do something like that that’s good for you to kind of manage clinical alerts.

Clinician 2

##### Cost

Most of the costs associated with implementing the *Medly* program were related to the equipment, which were perceived as high and unsustainable. However, plans are being made to reduce costs by having patients use their own devices. The other major cost involved the THA’s time, which was higher than initially anticipated.

Currently the system is CAD $2200 a pop for the phone, the data plan, the weight scale and the cuff so obviously, we don’t yet have a mechanism to pay for that and thankfully through [philanthropy] we’ll be able to cover the costs of the program.

Clinician 1

I think with time, we can drive the cost of [equipment] down. As we move to “bring your own device,” the phone costs [will] matter less... Where I think we’ve gone over budget [is that] we had anticipated that [telehealth] support would cost 25% of a person’s time and I think it’s coming closer to 50-75% of their time...and that overflows into the rest of the teams.

eHealth 2

This construct received a ranking of –1 because although the equipment cost was not a large initial barrier due to philanthropic funding, it was a barrier to program sustainability that would need to be addressed. Furthermore, although the additional THA time requirements did not significantly impact the *Medly* implementation, it did have important opportunity costs related to that individual’s ability to work on other projects. A formal economic evaluation of the impact of the *Medly* program from the perspectives of patients, HF clinic, and public payer will be published subsequently.

#### Outer Setting Domain

##### Patient Needs and Resources

This construct represents the idea that the implementation site comprehends and seeks to address patients’ needs. Numerous examples of the HF clinic’s patient-centered approach to care made this a strong facilitator.

It's a very supportive environment for patients and families, and that is something that I repeatedly hear in clinic, especially patients who have been in the clinic for a long period of time, how thankful and how grateful they are for the care that they receive. You hear this a lot, people just…they don’t want to go somewhere else.

Clinician 2

##### External Policy and Incentives

This construct had a neutral impact because although the program was perceived as compatible with government policies seeking to encourage more comprehensive chronic disease care outside of the hospital, factors like regulation, funding, and clinician reimbursement were flagged as crucial barriers to the program’s sustainability.

Any tool that we can develop that will actually improve patient-centred care…and potentially impact communication between different members of the team, which is the ultimate goal of Medly…are all in line with where the ministry of health is taking us.

Clinician 1

#### Inner Setting Domain

##### Networks and Communication

Three networks of communication were identified as having an influence on the implementation success. First, communication within the HF clinic was described as generally good and had a positive influence. Second, communication between clinicians and the THA involved in the day-to-day operations of the *Medly* program was also perceived as positive for the implementation. Finally, communication among high-level stakeholders was described as an early barrier to the implementation, particularly as it is related to decision making about the program and having clear channels to operationalize those decisions. This barrier was identified and notable improvements were made by the 4-month time point.

In the past, I think it was every possible channel…There were emails [that] didn't always go to the same people…Everyone knows what happens in the meetings, but it's what happened outside of those meetings where I think things were a lot more confusing. In addition to that, there was a lot of back-channel communication happening, and by that I don't mean between us, but I think between the stakeholders themselves…and then the rest of us eventually figure it out. So it was all over the place. And right now, I think it's consolidated a lot better.

eHealth 2

##### Culture

The HF clinic’s culture of teamwork was perceived as having facilitated both the implementation and the daily operations of the *Medly* program.

This hospital is like working in a 5-star hospital…we are a multidisciplinary team, so there are many people taking care of our patients, it’s not just us. It’s fantastic, it’s excellent. We are very patient-centred. In general, the environment or the mood in the clinic is positive and constructive.

Clinician 8

##### Implementation Climate

###### Tension for Change

Clinician participants were proud of the quality of care they offered to patients. However, a perceived gap existed between the current and ideal state. Coupled with a busy clinic with limited staff and space resources, this created a tension for change.

[Clinic capacity] is an ongoing concern for me and I think we're at a bit of a crux where we couldn’t handle somebody not coming to work and we can't handle any more volume.

Clinician 1

###### Compatibility

The *Medly* program was perceived as compatible with the values of the organization and complimentary to existing services offered in the HF clinic.

Offering patients something different and unique that is more based on technology that they can use at home, I think totally fits with UHN’s goals and vision with advancing patient care…I don’t see anything else that we’re doing that overlaps with what Medly’s doing. I mean, one of the things that we want to try and do a lot more of is education in the clinic environment for patients and I think, if anything, Medly just completely supports those messages that we give to patients about why salt restrictions are important and those kinds of things. So I don’t see it as a duplication, I think it just kind of nicely fits together in terms of more comprehensive care for patients.

Clinician 2

Early apprehension about increased clinician workloads speaks of the incompatibility of the telemonitoring program with existing clinic workflows. However, by the end of the first year, evidence exists that a new normal has been created such that this initial incompatibility did not significantly impact the overall implementation success.

I organize my time differently now…I've changed the way I do things because I can’t be in clinic doing clinic and trying to run back and forth because that’s challenging. So, I try to carve out like at least the first half an hour or hour of my day to deal with Medly and then I go [to clinic].

Clinician 3

###### Relative Priority

The implementation of the *Medly* program was perceived as having a high priority by all participants.

My understanding is that Medly is a fairly high priority…A lot of the other [initiatives] are still important and they’re going on simultaneously, but I would say [Medly]’s up there.

Clinician 3

###### Learning Climate

Participants describe a work environment that values ongoing quality improvement. They feel the climate offers a safe space for learning and trying new things, making this construct a strong positive influence on implementation success.

I work with a great staff, very closely with a few heart failure physicians who have been fantastic in advancing my knowledge and teaching me along the last one year.

Clinician 2

##### Readiness for Implementation

###### Available resources

Important to the success of this implementation was the availability of financial and human resources. No new clinicians were employed; rather, existing NPs were expected to perform *Medly*-related tasks within their salaried hours. Although this was possible, the added NP workload should not be underestimated.

[I am] not complaining about [responding to alerts] because that is part of why I'm hired. It’s just that there needs to be, in order for Medly to work, you have to have a clinician who is devoting time to do all of that, to answer alerts, to document, and to see patients that are unwell in clinic.

Clinician 3

The THA was an additional resource that was hired to support this program. Flexibility with respect to this resource, both in terms of quantity of time and time during the day, was an important facilitator that might not be realistic in other sites.

It makes it a lot easier when they call me down to the clinic or they have a patient come to the clinic and I am available and I can just run down and be there in five minutes. My worry would be if it was a different site and they need that kind of instant support. It may be difficult getting someone there.

eHealth 4

Funding for the equipment came from philanthropic donations, thereby mitigating the potential barrier of nonavailability of funds common in many real-world implementations.

The cost, although improved, is still an issue, because right now Medly is being funded [by philanthropy] and obviously, we're not here to fund it for the province.

Clinician 1

Finally, insufficient physical space is a challenge for the HF clinic and was likely an indirect barrier as clinic rooms are required for patient onboarding.

What hasn’t been solved is the fact that there aren’t enough resources in terms of rooms, in terms of workflow around patients getting seen and into the rooms, we’re limited by the physical space.

Clinician 7

###### Access to Knowledge and Information

The availability of the THA to provide on-call and personalized information about how to use the intervention was an important facilitator. However, although clinicians perceive the training they received to be sufficient, some felt that more comprehensive training around understanding the algorithm was required. In addition, the novelty of this program meant that no clear medical-legal guidelines existed on exactly what information needed to be documented. Therefore, this is a challenge that clinician users needed to navigate on their own.

That was a little bit confusing maybe [for us], what should be documented in terms of alerts and what should not. So that's kind of just been teased out as we've been going through it for the last four months.

Clinician 2

#### Characteristics of Individuals Domain

##### Knowledge and Beliefs About the Intervention

Clinician knowledge and positive beliefs about the *Medly* program likely helped overcome the potentially negative influence of the equivocal scientific evidence.

I don't know if I’m just being an optimist. I actually think [the Medly program] is going to show reduced hospital length of stay and admissions. And so I think that if the system has proven to do this, I think it’s going to be useful across the board because heart failure is everywhere.

Clinician 4

##### Self-Efficacy

Despite initial apprehensions about increased workload, the clinician and eHI teams were confident that they would be successful in implementing the program.

I don’t think that there’s any doubt that we will be implementing it I think as intended. While I may be apprehensive, it doesn’t mean that I don’t think that we still actually need to try and actually see.

Clinician 3

#### Process Domain

##### Planning

Despite user training being planned and the initial service design work leading up to the program launch, an overarching implementation plan was never explicitly developed at the outset; this was perceived as having a negative impact during the initial months of the implementation. However, after realizing this deficiency, ongoing plans were formalized by the team; this was perceived as having a positive influence on the current and future program.

Since the four-month, we regrouped as an op[eration]s team…I think we have a much better strategy for what we’re trying to do and we actually now have people dedicated to that…I think there’s a much more coherent strategy and a much more coherent plan.

eHealth 2

##### Engaging Champions and Opinion Leaders

The presence of a clinician champion or opinion leader was an important facilitator for both the development and implementation of the *Medly* program. Importantly, the fact that this champion and opinion leader set a positive example appears to have had more of an impact on the implementation success than this individual’s role as a formal leader.

I think certainly that from the clinic side, that [Clinician 1] is the champion of this. She’s pushed very hard for its development and rollout and by far I think she’s certainly enrolled the largest number of patients onto the system.

Clinician 3

##### Executing

Although no formalized implementation plan existed, overall, there is a perception that the eHI team has been effective in doing what was necessary to support the implementation. However, the team’s inability to delivery rapid technology adaptations was perceived as a barrier by all participants.

I think there have been some deviations, but overall, I think the team is doing a relatively good job with meeting the expectations. I think some of the deviations are reasons outside of our control or some of them are just because of delays in development. I know a lot of the things we want to do with the program around streamlining it involve adapting the technology and we haven’t been able to fully do that. But on the process side, we’ve been responding pretty well.

eHealth 2

##### Reflecting and Evaluating

Embedded within the *Medly* program was a mechanism to facilitate the ongoing quality improvement. All participants spoke positively of the benefits of being able to quickly identify and evaluate problems and implement solutions when possible.

[We meet] every two weeks to discuss the recruitment in the program, how things are going, any issues or problems that people have faced. And then we discuss those issues, identify solutions and come up with a plan for how we want to address them. We also talk about achievements that have happened, so recruitment milestones, things like that.

eHealth 2

## Discussion

### Principal Findings

This longitudinal implementation evaluation found that the *Medly* program had been successfully implemented, as demonstrated by the steady growth in patient enrollment and clinician adoption and the fidelity with which the intervention was being used in clinical practice. Costs were relatively high because of the decision to initially supply patients with all the telemonitoring equipment. That said, these costs were not estimated to have significantly impacted the implementation and are expected to dramatically decrease, as the program shifts to a bring your own device (BYOD) model. This study also identified 24 CFIR constructs that explain these measures of implementation success. Fifteen constructs were facilitators predominately clustered in the domains of *inner setting* and *characteristics of individuals*. Four CFIR constructs were minor barriers in the earlier phases of implementation—*complexity*, *cost*, *networks and communication*, and *planning*. Five additional constructs had a mixed valence and therefore were determined to have a neutral impact on the implementation.

### Comparison with Prior Work

The implementation barriers and facilitators identified in this study are very much in line with results from other telemonitoring implementation studies. Systematic reviews have concluded the importance of characteristics of the technology, people involved, extraorganizational environment, and implementation setting [11-13]. In addition, the literature suggests that having undefined roles in the context of new workflows is a common barrier to eHealth implementations [16]. This study provides concrete examples of these barriers as they relate to the CFIR constructs of *complexity* and *compatibility*. For example, in the absence of clear guidelines for documentation, identifying ideal patient candidates and setting parameter thresholds needed for the algorithm, clinicians are forced to develop experiential knowledge to be able to perform these tasks. The development of this tacit knowledge can often only happen over time and might be challenging in a fast-paced clinic environment. The learning climate in this study was perceived as being an important facilitator, which likely helped overcome this challenge. However, clear guidelines for clinician staff roles will likely be required to ensure implementation success where learning climates might not be as favorable.

In addition to providing a framework that allows for the easier transferability of study results, using CFIR-guided methods allowed this research to make two additional contributions to the field of implementation science. To the best of our knowledge, this is the first study to demonstrate the feasibility of using the CFIR for evaluating complex telehealth interventions [24]. However, the CFIR lacks granularity for identifying factors that might be unique to health information systems. Researchers wanting an in-depth understanding of the impact of the technology (as opposed to the full intervention) should consider informing their methods using an additional framework that will help open the black box of *design quality and packaging*. For example, the Clinical Adoption Framework could be useful for designing probes around the quality of the system, quality of the information within the system, and quality of the services supporting the system [25]. Another limitation of the CFIR is that we consider most software updates to be an inherent quality of software-based health interventions. As such, we do not think that a technology’s capacity to iterate is adequately captured in the CFIR construct of *adaptability*, which relates more to the components of an intervention that can be adapted or tailored.

Unlike studies that present a list of barriers and facilitators, the CFIR guides the classification of these factors into broader domains. For example, the strongest influencing factors on the *Medly* program were in the CFIR domains of *inner setting*, *characteristics of individuals*, and *process*. This is not to say that the characteristics of the intervention were not important, but it makes the point that the implementation context cannot be ignored.

### Limitations

This study was conducted at a single implementation site. Therefore, we acknowledge that the characteristics of the HF clinic might differ compared with other settings in terms of the availability of resources, structure of care delivery, and characteristics of the individuals involved. In addition, we acknowledge the absence of the patients’ perspective in this study. That said, a mixed method study of factors that influence patient adoption, use, and adherence to the *Medly* program will feature in a future publication. Finally, one cardiologist did not agree to participate in an interview; therefore, barriers to adoption for this individual are unknown.

### Recommendations

We offer the following recommendations based on key study findings to facilitate the transferability of results to other implementation settings:

1. Evaluate contextual barriers: This study highlights the importance of contextual factors. Early identification of potential barriers as part of a readiness assessment would allow for the development of mitigating strategies. Using a framework such as the CFIR could facilitate this task; our results provide an example of how the CFIR constructs can be operationalized for telehealth interventions.
2. Define all components of the intervention: Complexity is an important barrier for the successful implementation of eHealth interventions [16]. However, this negative influence can be mitigated by an explicit definition of each intervention component. In this study, contextual facilitators helped overcome the lack of protocolized clinician roles; however, better definition of nontechnology program components and roles could facilitate clinician adoption in future implementations.
3. Plan and document the implementation strategy: The lack of clearly defined implementation plan was identified as an early barrier in this study, which was moderated by contextual facilitators and other *process* factors, including the presence of a strong clinical champion and a robust mechanism for ongoing reflecting and evaluation. The Quality Implementation Framework [26] offers a prescriptive approach that can help formulate an implementation strategy that incorporates an assessment of many of the constructs outlined in the CFIR [19].

### Conclusions

This study presents results from the real-world implementation evaluation of a mobile phone–based telemonitoring program for patients with heart failure. The overall success of the implementation, as determined by the four implementation outcomes, was explained by the presence of several facilitators and relatively few barriers. Although the results are consistent with other telemonitoring implementation studies, this study also demonstrates how barriers and facilitators are dynamic and can influence the implementation success differently over time. Finally, we highlight a previously undescribed challenge—telemonitoring interventions often rely on clinicians’ ability to build experiential knowledge to use the system as intended. The results from this research can inform the development of telemonitoring programs and their implementation strategies. Hence, evidence-based implementation is important to ensure the success of real-world telemonitoring deployments as well as for ensuring that telemonitoring studies can yield unambiguous evidence of effectiveness, which will be required for the wider diffusion of telemonitoring.

## Abbreviations

BYOD: bring your own device
CFIR: Consolidated Framework for Implementation Research
eHI: Centre for Global eHealth Innovation
EMR: electronic medical record
HF: heart function
NP: nurse practitioner
TM: telemonitoring
THA: telehealth analyst
UHN: University Health Network
